# Arts-Based Interventions for Dementia Care: East Meets West Symposium

**DOI:** 10.1093/geroni/igaa057.1871

**Published:** 2020-12-16

**Authors:** Fei Sun, Angel Duncan, Nancy Hooyman

## Abstract

This East Meets West symposium presents evidence of arts-based interventions in dementia care in different societal settings, focusing on the U.S.A. and China, where live about one-third of the world’s total estimated 49 million dementia population. The first study from Kansas in the U.S. outlined the varieties of arts being applied in dementia care and recommended dementia care, inter-professional teams, to involve those professionals in arts and humanities. The second paper, based upon secondary national representative data, examined the association with arts-related hobbies and cognition status among Chinese older adults. The authors called for more research to shed light on the underlying mechanisms between arts and cognition. The third paper discussed two arts-based clinical trials on persons with dementia (PWD) at different stages living in Hong Kong. It found that dancing body movement therapy improved behavioral and emotional outcomes among those at the mild dementia stage. In contrast, music and movement worked better for those at the moderate dementia stage. The fourth study reviewed the effectiveness of body movement therapies for PWD, using an example of the Wheelchair-bound Senior Elastic Band for older adults with disability and dementia. The last study examined the effectiveness of a program that used museum tours to empower, educate, and inspire PWD. One discussant will share lessons learned across studies, and the other discussant from AARP Global Council on Brain Health will speak to the effects of music relating to the AARP 2020 consensus report on music and the brain health.

